# Native Rhizobia Improve Plant Growth, Fix N_2_, and Reduce Greenhouse Emissions of Sunnhemp More than Commercial Rhizobia Inoculants in Florida Citrus Orchards

**DOI:** 10.3390/plants11223011

**Published:** 2022-11-08

**Authors:** Antonio Castellano-Hinojosa, Christoph Mora, Sarah L. Strauss

**Affiliations:** Southwest Florida Research and Education Center, Department of Soil, Water, and Ecosystem Sciences, Institute of Food and Agricultural Sciences, University of Florida, 2685 State Rd. 29N, Immokalee, FL 34142, USA

**Keywords:** legumes, sunnhemp, cover crops, orchards, citrus, biofertilizers, denitrification, nodulation, nitrous oxide

## Abstract

Sunnhemp (*Crotalaria juncea* L.) is an important legume cover crop used in tree cropping systems, where there is increased interest by growers to identify rhizobia to maximize soil nitrogen (N) inputs. We aimed to isolate and identify native rhizobia and compare their capabilities with non-native rhizobia from commercial inoculants to fix atmospheric dinitrogen (N_2_), produce and reduce nitrous oxide (N_2_O), and improve plant growth. Phylogenetic analyses of sequences of the 16S rRNA and *recA*, *atpD,* and *glnII* genes showed native rhizobial strains belonged to *Rhizobium tropici* and the non-native strain to *Bradyrhizobium japonicum*. Plant nodulation tests, sequencing of *nodC* and *nifH* genes, and the acetylene-dependent ethylene production assay confirmed the capacity of all strains to nodulate sunnhemp and fix N_2_. Inoculation with native rhizobial strains resulted in significant increases in root and shoot weight and total C and N contents in the shoots, and showed greater N_2_-fixation rates and lower emissions of N_2_O compared to the non-native rhizobium. Our results suggest that native rhizobia improve plant growth, fix N_2_, and reduce greenhouse emissions of sunnhemp more than commercial rhizobia inoculants in Florida citrus orchards.

## 1. Introduction

Cover cropping is a common agricultural practice and there is extensive research documenting its benefits in improving soil nutrient cycling, pest management, and agriculture production, and reducing soil erosion and weed pressure [1,2,3]. In annual crops, cover crops (CCs) are grown primarily between periods of regular crop production [2]; however, in fruit tree crops, CCs are grown in the inter-row middles [4,5].

In both annual and tree cropping systems, legumes are often part of CC mixtures. Legumes are a large group of angiosperms that comprise more than 20,000 species and 750 genera, are found on all continents, and can grow in diverse edaphic and climatic conditions [6]. One of the key reasons legumes are used in CC mixtures is to increase the soil nitrogen (N) pool through their ability to establish symbiotic associations with diazotrophic bacteria, commonly known as rhizobia. These bacteria induce the formation of root nodules where atmospheric dinitrogen (N_2_)-fixation takes place, conferring upon legumes an important ecological advantage [6]. Rhizobia are phylogenetically diverse both in core and symbiotic gene sequences and new species are described every year [6,7]. In addition to rhizobia, other endophytic bacteria are also often found in legume nodules, and recent studies have shown their possible essential role in plant growth [8,9].

The specificity, infectivity, and effectiveness of rhizobia in the symbiotic relationship with a legume are due to the expression of different molecules by the bacterium, the host plant, or both [6]. Whereas some legumes can be nodulated by several rhizobial species such as *Phaseolus* or *Macroptilium*, others such as *Cicer* spp. or *Trifolium* spp. are restrictive hosts for nodulation [6]. In addition, rhizobial strains can have a narrow or a broad host range and their ability to nodulate multiple legume species depends on the presence of different nodulation genes in their genome and the legume promiscuity [6]. The nodulation genes (*nod)* are involved in the synthesis of Nod factors that are plant flavonoid receptors [6,10]. Within *nod* genes, the *nodC* is commonly used to analyze the host range of rhizobia and the promiscuity degree of the hosts [6,11,12].

In addition to the agronomic interest in identifying effective N_2_-fixing rhizobia, extensive research has been conducted on the ability of these microorganisms to produce and reduce nitrous oxide (N_2_O), an ozone-depleting substance and important greenhouse gas [13]. Previous studies have shown that many legume-nodulating rhizobacteria do not perform complete denitrification [14], the process by which nitrate (NO_3_^−^) is sequentially reduced to N_2_O and finally N_2_ under oxygen-limiting conditions [15]. Inoculating crop legumes with rhizobia not only capable of fixing N_2_ but also of reducing N_2_O emissions is therefore of great environmental and agronomic interest in order to increase the sustainability of agriculture [14,16].

In both annual and tree cropping systems, growers often inoculate seeds of legume CCs with commercial rhizobia to ensure successful nodulation, and therefore effective N_2_-fixation. These products are often presented as a sustainable and cost-effective technology to maximize N inputs from legumes [17], and previous studies have shown that inoculation with rhizobia can improve legume growth and productivity [18,19]. However, inoculation with commercial inoculants does not always give positive yield results when the inoculated non-native rhizobia are not adapted to the environmental conditions and/or soil characteristics of the region [20,21]. In other cases, the inoculants do not contain rhizobia capable of effectively nodulating the legume of interest and/or they are less effective in nodulating or fixing N_2_ than native rhizobia [21]. Native rhizobia have been shown to better nodulate legume plants compared to commercial inoculants [22], and there has been extensive research on the isolation and characterization of native rhizobia as an alternative to use of commercial rhizobia inoculants [23,24].

*Crotalaria* is a genus of tropical legumes that comprises more than 550 species [25]. Sunnhemp (*Crotalaria juncea* L.) has been grown as a green manure crop for centuries in tropical and subtropical regions worldwide. Recent studies have shown that sunnhemp can be an effective CC in subtropical fruit tree crops such as citrus in Florida, where it can be planted year-round in the inter-row middles [26]. While *C. juncea* can establish symbiotic associations with rhizobia belonging to the genera *Bradyrhizobium* [27,28] and *Methylobacterium* [29] in West Africa, less is known about the rhizobial species nodulating sunnhemp in other regions. Field observations by growers in Florida citrus orchards have shown contrasting results in the effectiveness of commercial inoculants to nodulate sunnhemp. Therefore, the objectives of this study were to: (i) isolate and identify the native rhizobia strains nodulating sunnhemp from soils of two different commercial citrus orchards in Florida and isolate and identify rhizobia from a commonly-used inoculant, (ii) evaluate the ability and effectiveness of native vs. non-native rhizobial strains to fix N_2_ and produce and reduce N_2_O, and (iii) determine the effect of inoculating sunnhemp plants with native vs. non-native rhizobial strains on root and plant growth, N_2_ fixation rates, and N_2_O and N_2_ emissions from nodules under greenhouse conditions.

## 2. Results

### 2.1. REP-PCR, 16S rRNA, recA, glnII, and atpD Phylogenetic Analyses

A total of 47 CFUs were isolated from root nodules of sunnhemp plants. Out of the 47 bacterial strains, 13, 19, and 15 were isolated from plants inoculated with soil from citrus orchard A (COA), citrus orchard B (COB), and the commercial rhizobial inoculant (MI), respectively. After REP-PCR fingerprinting, the 47 isolates were represented by 26 different profiles at 80% similarity (Table 1). The nearly complete sequence of the 16S rRNA gene from a representative strain of each REP group showed that they were members of the genera *Bacillus* (12 strains), *Rhizobium* (4 strains), *Burkholderia* (3 strains), *Paenibacillus* (3 strains), *Herbaspirillum* (2 strains), *Paenarthrobacter* (1 strain), and *Bradyrhizobium* (1 strain) (Table 1). Further characterization of strains associated with non-rhizobia genera was not pursued in this study. Phylogenetic analyses of *Rhizobium* and *Bradyrhizobium* strains were conducted separately to better account for species differences among the members of each genus.

**Figure 1:**
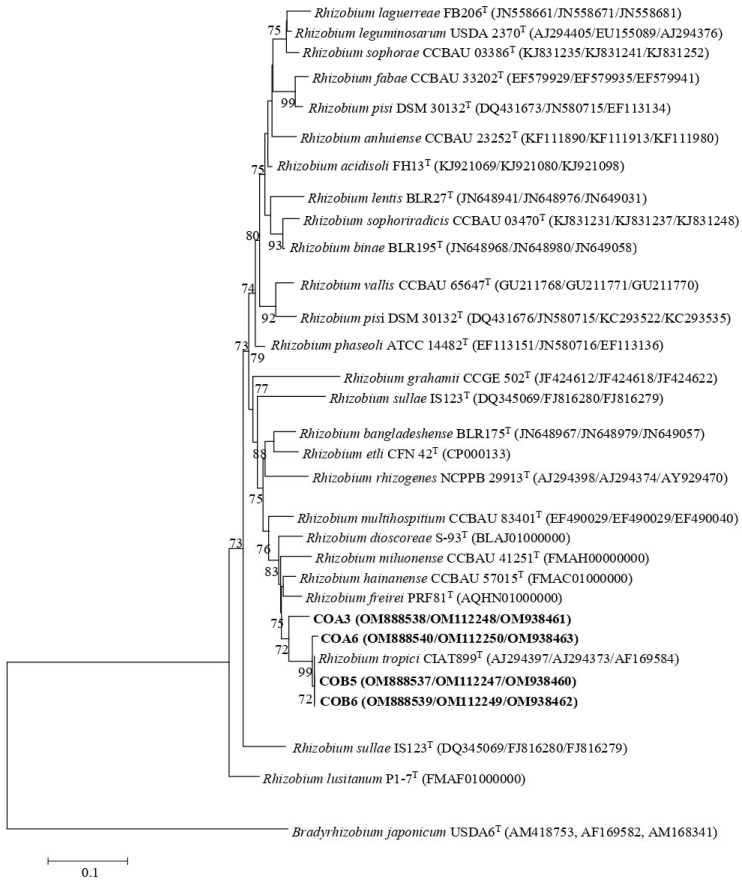
ML phylogenetic tree based on concatenated partial *recA* + *atpD* + *glnII* sequences of strains from nodules of sunnhemp (*Crotalaria juncea* L.) and phylogenetically related species within the genus *Rhizobium*. The analysis was based on 1150 nucleotides. Isolates are denoted in bold. Bootstrap values are indicated as percentages derived from 1000 replications. Values lower than 70 are not shown. Bar, 1 nucleotides substitution per 100 nucleotides. The tree is rooted with *Bradyrhizobium japonicum* USDA6^T^.

**Figure 2:**
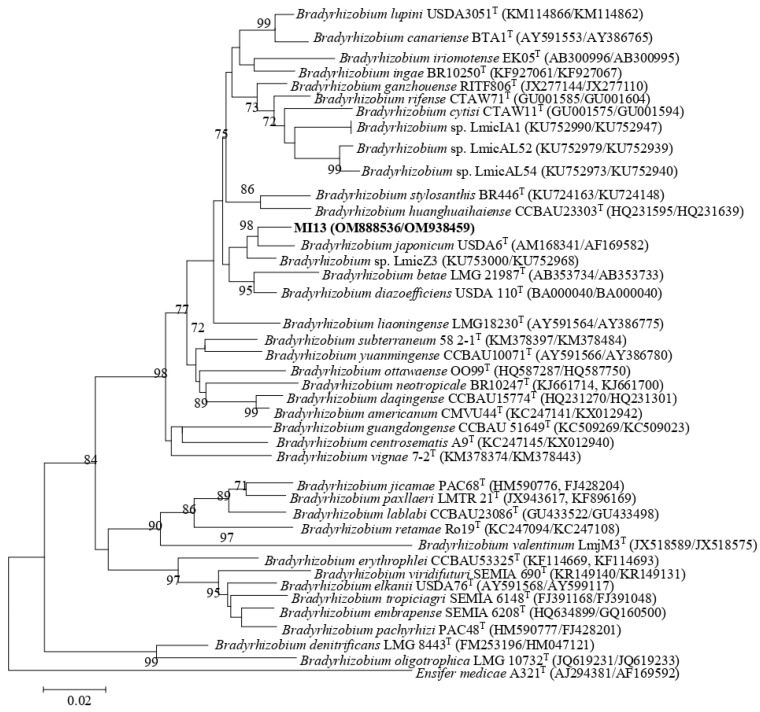
ML phylogenetic tree based on concatenated partial *recA* + *glnII* sequences of strains from nodules of sunnhemp (*Crotalaria juncea* L.) and phylogenetically related species within the genus *Bradyrhizobium*. The analysis was based on 870 nucleotides. Isolates are denoted in bold. Bootstrap values are indicated as percentages derived from 1000 replications. Values lower than 70 are not shown. Bar, 2 nucleotides substitution per 100 nucleotides. The tree is rooted with *Ensifer medicae* A321^T^.

The phylogenetic tree inferred from the 16S rRNA gene sequences revealed that the strains COA3, COA6, COB5, and COB6 showed identity values higher than 99.0% with *R. tropici* CIAT 899^T^, *R. freirei* PRF 81^T^, and *R. hainanense* CCBAU 57015^T^ (Supplementary Figure S2; Supplementary Table S2). Strain MI13 had 99.85% and 99.93% identity with *B. japonicum* USDA6^T^ and *B. liaoningense* LMG18230^T^, respectively (Supplementary Figure S3; Supplementary Table S2). Due to difficulties in assigning species to the genera *Rhizobium* and *Bradyrhizobium* with only sequences of the 16S rRNA gene, the *recA*, *atpD*, and *glnII* housekeeping genes were also sequenced. A concatenated phylogenetic tree based on the obtained *recA*, *atpD,* and *glnII* sequences revealed that strains COA6, COB5, and COB6 grouped with *R. tropici* CIAT 899^T^ with identity values higher than 99.6% (Figure 1; Supplementary Table S2). Strain COA3 also clustered with *R. tropici* CIAT 899^T^ but with identity values lower than 96.7% (Figure 1; Supplementary Table S2). A concatenated phylogenetic tree based on only the *recA* and *glnII* sequences showed that strain MI13 was closely related to *B. japonicum* USDA6^T^ with 99.5% identity (Figure 2; Supplementary Table S2).

### 2.2. nodC and nifH Phylogenetic Analyses

**Figure 3:**
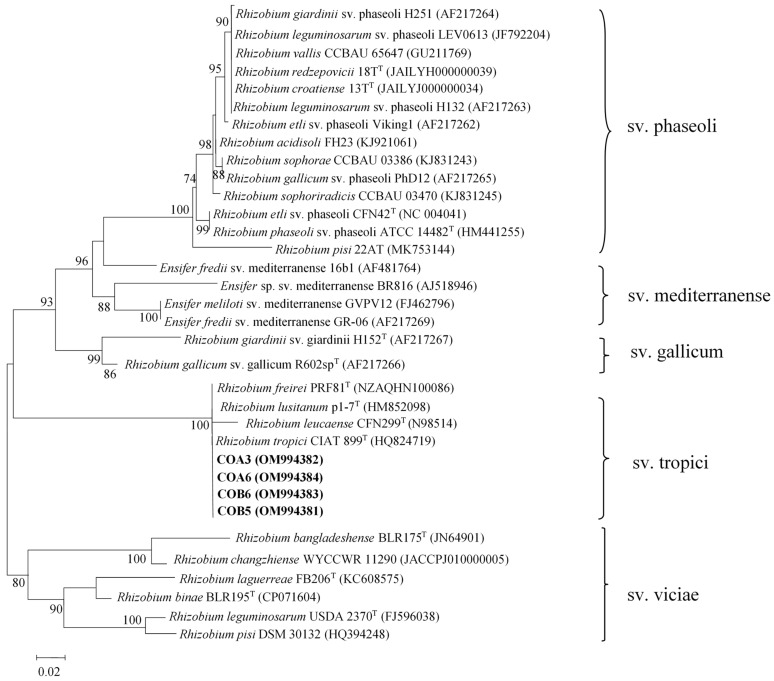
ML phylogenetic tree based on *nodC* sequences of strains from nodules of sunnhemp (*Crotalaria juncea* L.) and phylogenetically related species within the genus *Rhizobium*. *Rhizobium* symbiovars are also shown. The analysis was based on 580 nucleotides. Isolates are denoted in bold. Bootstrap values are indicated as percentages derived from 1000 replications. Values lower than 70 are not shown. Bar, 2 nucleotide substitution per 100 nucleotides.

**Figure 4:**
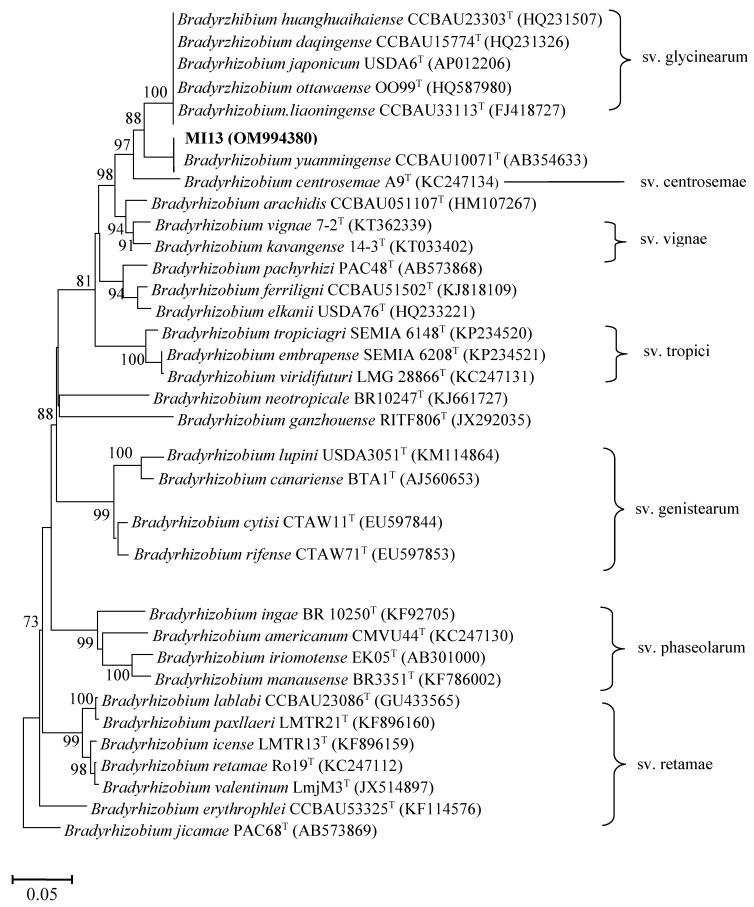
ML phylogenetic tree based on *nodC* sequences of strains from nodules of sunnhemp (*Crotalaria juncea* L.) and phylogenetically related species within the genus *Bradyhizobium*. *Bradyhizobium* symbiovars are also shown. The analysis was based on 580 nucleotides. Isolates are denoted in bold. Bootstrap values are indicated as percentages derived from 1000 replications. Values lower than 70 are not shown. Bar, 5 nucleotide substitution per 100 nucleotides.

A phylogenetic tree showing the relationship between the *nodC* genes from COA3, COA6, COB5, and COB6 strains and other rhizobial species revealed that all four strains belonged to symbiovar tropici of the genus *Rhizobium* with identities higher than 99% (Figure 3; Supplementary Table S2). Strain MI13 formed a cluster with *B. yuanmingense* CCBAU10071^T^ based on the sequences of the *nodC* gene with 99.9% identity (Figure 4; Supplementary Table S2). This cluster is phylogenetically divergent to that formed by the *Bradyrhizobium* symbiovars defined to date (Figure 4).

The phylogenetic trees inferred from sequences of the *nifH* gene revealed that strains COA3, COA6, COB5, and COB6 grouped with *R. tropici* CIAT 899^T^, *R. multihospitium* CCBAU 83401^T^, and *R. lusitanum* P1-7^T^ with identity values higher than 99% (Supplementary Figure S4; Supplementary Table S2), and that the strain MI13 clustered with *B. daqingense* CCBAU 15774^T^, *B. huanghuaihainense* CCABU 23303^T^, *B. ottawaense* OO99^T^, *B. liaoningense* LMG 18230^T^, and *B. japonicum* USDA6^T^ with identity values of 99.9% (Supplementary Figure S5; Supplementary Table S2).

### 2.3. N_2_-Fixation Rates and Nitrous Oxide and Dinitrogen Emissions from Free-Living Cells and Nodules

Nodules from the trapping experiment showed differences in N_2_-fixation rates and N_2_O emissions between plants inoculated with soils from COA, COB, and the MI. N_2_-fixation rates were significantly greater from plants inoculated with COA and COB compared to the MI (Supplementary Table S3). Significantly greater N_2_O emissions were detected from nodules of plants inoculated with the MI compared to COA and COB (Supplementary Table S3) but no significant differences in N_2_ emissions were detected between treatments (Supplementary Table S3A).

In free-living cells, strains COA3 and COA6 showed significantly greater N_2_-fixation rates compared to strains COB5, COB6, and MI13 (Table 2A). All strains were able to grow under denitrification conditions with NO_3_^−^ as the sole N source, and production of N_2_O and N_2_ evidenced their ability to metabolize NO_3_^−^ (Table 2A). Strain MI13 showed significantly greater N_2_O production rates compared to strains COA3, COA6, COB5, and COB6 (Table 2A). No significant differences in N_2_ production rates were detected between strains (Table 2A).

Nodules from roots of sunnhemp plants inoculated with strains COA3 and COA6 showed significantly greater N_2_-fixation rates compared to the rest of the strains (Table 2B). Nodules from plants inoculated with MI13 were the highest N_2_O producers compared to those of COA3, COA6, COB5, and COB6, whereas no significant differences in N_2_ emissions were detected between strains (Table 2B).

### 2.4. Plant Nodulation and Inoculation Assays

Strains COA3, COA6, COB5, COB6, and MI13 produced effective symbiosis with sunnhemp plants and REP-PCR confirmed the presence of the inoculated bacteria in the root nodules of the corresponding inoculated plants. The results of plant inoculation assays showed that all strains except for MI13 significantly promoted the growth of sunnhemp with respect to the uninoculated control plants (Table 3). Inoculation of sunnhemp with strains COA3 and COA6 produced significant increases in the number of nodules, root and shoot dry weight, and total N and C concentrations compared to strains COB5, COB6, and MI13 (Table 3). The inoculation of sunnhemp with COB5 and COB6 strains significantly improved the root and shoot dry weight and the concentration of total N and C in the shoots with respect to strain MI13 (Table 3).

## 3. Discussion

The low soil nutrient availability of many tree crops has renewed interest in the use of CCs in row middles as an alternative to conventional management (e.g., spontaneously growing weeds or no vegetation) to improve soil C and N cycling for these agroecosystems [5,30]. Sunnhemp is an important CC in subtropical fruit tree crops such as Florida citrus due to its ability to grow year-round, but no studies have examined the diversity of rhizobia nodulating this plant in these agroecosystems. Our study showed that native Florida rhizobia are better at nodulation, N_2_ fixation, reducing N_2_O emissions, and increasing plant growth compared to non-native rhizobia from a commonly-used commercial inoculant. Although all isolated native rhizobia were grouped with the same *Rhizobium* species, we showed they had different abilities to nodulate, fix N_2,_ and produce and reduce N_2_O, suggesting that these traits were strain-specific. To our knowledge, this is the first report on strains of *Rhizobium tropici* nodulating *C. juncea*, thus increasing our understanding of the diversity of rhizobia nodulating *Crotalaria* in Florida soils. The benefits of native over non-native rhizobia were not only found under laboratory conditions but also under symbiotic conditions when plants were inoculated with the strains using sterile and non-sterile substrates, thus confirming the potential of native rhizobial strains to be used for future microbial-based inoculants.

Florida soils have an extremely sandy texture (>90% sand) and very low content of C and N, which can cause severe ecological conditions for living organisms [31]. REP fingerprinting, a powerful tool to cluster rhizobia at the subspecies and strain level [32], was used to group the isolates from root nodules. Only 5 of the 47 isolates were identified as rhizobia. The rest of non-rhizobial isolates were bacterial endophytes which are often present in root nodules of leguminous plants and may have key roles in the promotion of legume growth [9]. Interestingly, based on the 16S rRNA gene sequences, all native rhizobia (four strains) belonged to *Rhizobium,* whereas the non-native rhizobium isolated from the microbial inoculant was identified as a member of *Bradyrhizobium*. Due to highly conserved 16S rRNA gene sequences in rhizobia, the phylogenetic analysis of *recA*, *atpD*, and *glnII* housekeeping genes have been used to elucidate the taxonomic affiliations of *Rhizobium* and *Bradyrhizobium* species. While the four native rhizobia grouped with *R. tropici* CIAT 899^T^, the non-native strain was closely related to *B. japonicum* USDA6^T^.

All rhizobial isolates produced effective symbiosis with sunnhemp plants. Previous studies have shown that *C. juncea* establishes symbiotic associations with rhizobia belonging to *Bradyrhizobium* [27,28] and *Methylobacterium* [29] genera in West Africa. Strains of *Rhizobium* sp. have been previously isolated from root nodules of *Crotalaria* species but were unable to nodulate this plant [33]. To our knowledge, this is the first report on strains of *R. tropici* nodulating *C. juncea,* and the sequencing of the *nodC* gene revealed that all native rhizobial strains belonged to symbiovar tropici of the genus *Rhizobium*. The symbiovar tropici is composed of strains of *R. tropici*, *R. leucaenae*, *R. lusitanum,* and *R. freirei*, and has been found in America, Africa, and Asia [34,35]. *R. tropici* has a broad host range and can nodulate several legumes including *P. vulgaris*, *Leucaena leucocephala,* and *Macroptilium atropurpureum* due to the existence of different *nod* genes in its genome [6]. In the tropics, inoculants containing *R. tropici* are efficient for beans due to its high tolerance to stressful conditions and genetic stability [36,37]. Of note, the concatenated *recA*, *atpD,* and *glnII* gene phylogenies showed that strain COA3 had similarity values lower than 97% with *R. tropici* CIAT 899^T^ and could represent new lineages within the genus *Rhizobium*. However, this should be further studied by conducting a polyphasic taxonomic analysis.

Previous studies have shown that *Crotalaria* sp. can be nodulated by different species of *Bradyrhizobium* including *B. japonicum* [28,33,38,39]. The phylogenetic analysis of the symbiotic *nodC* gene of the non-native strain MI13 revealed that it clustered outside of known symbiovars of the genus *Bradyrhizobium* and was closely related with *B. yuanmingense*. Strains of *B. yuanmingense* can nodulate the wild legume *Lespedeza* and form ineffective symbiotic associations with *Medicago sativa* and *Melilotus albus* [40].

Although both native and non-native rhizobia strains isolated in this study possessed *nifH* genes and therefore the potential to fix atmospheric N_2_, native rhizobia showed significantly greater N_2_-fixation rates than the non-native rhizobium both in free-living cells and symbiotic conditions. Although commercial rhizobial inoculants typically contain strains with excellent performance, the degree to which non-native rhizobial strains adapt to local environmental and soil conditions and competition with native rhizobia can directly affect their nodulation and N_2_-fixation effectiveness [21]. Strains of *R. tropici* and *B. japonicum* are known to possess *nifH* genes and the ability to fix N_2_ [34,41]. Among native rhizobia, those isolated from citrus orchard A had significantly greater N_2_-fixation rates compared to the ones from citrus orchard B. Although these two citrus orchards share similar environmental conditions, soils were slightly more fertile in citrus orchard A compared to orchard B (Supplementary Table S1) which may have influenced diversification between rhizobial strains in these Florida soils. Influences of soil environmental conditions on rhizobial diversity and N_2_-fixing capabilities have been previously reported for other rhizobia species [42,43,44].

Knowledge of the diversity and capabilities of plant-growth promotion of native bacterial populations is crucial to identify specific microbial strains that can be used to achieve higher yields under specific environmental conditions [45]. Previous studies have shown that native rhizobial populations may enhance plant-microbe interactions due to their better adaption to natural conditions [46]. Our results agree with this rationale by showing that the sunnhemp plants inoculated individually with native rhizobial strains had a significant increase in root and shoot dry weight and a concomitant enhancement of total C and N content compared to the non-native MI13 strain. Field inoculation studies are recommended to fully elucidate the potential of the isolates in this study to serve as candidates for future rhizobial inoculants.

We also found that native rhizobia emitted less N_2_O in free-living cells and symbiotic conditions compared to the non-native rhizobium strain. Denitrification can cause loss of N in soil and it is not a desirable characteristic of inoculant bacteria. It is known that not all rhizobial strains can grow under denitrification conditions [14,47,48,49]. However, all rhizobial strains isolated in this study were able to grow under these conditions and to produce N_2_O and N_2_ in free-living cells and symbiotic conditions. Although amplification of denitrification genes was not pursued in this study, previous studies have shown that both strains of *R. tropici* and *B. japonicum* possess genes involved in production (e.g., *nirK* and *nirS*) and reduction (*nosZ*) of N_2_O [41,48]. Because rhizobial species often lack the N_2_O reductase [14,50,51], there is increased interest in identifying rhizobia capable of emitting N_2_ as a final product. Considering the increased use of legume cover crops in tree crops, the legume–rhizobium symbiosis could play an important role in reducing N_2_O emissions, thus contributing to the alleviation of global warming [16]. Calculations of the N_2_O/N_2_ ratio showed values close to 1 for the native rhizobia strains both in free-living cells and under symbiotic conditions whereas those for the non-native rhizobium were in the range 2.5–3. Thus, these results suggest the native rhizobial strains isolated in this study represent excellent candidates that would increase soil N without contributing to production of N_2_O in citrus agroecosystems.

## 4. Conclusions

To date, most growers are aware of the ecological advantage of legume CCs to fix atmospheric N_2_, which is particularly important for high N-demanding crops in soils with low nutrient availability such as citrus in Florida. However, the specificity of the symbiosis between rhizobia and legume is often not known for specific legume species of interest. We determined the phylogeny of native rhizobial strains nodulating *C. juncea* in citrus orchards in Florida, a commonly used CC species in subtropical tree crops. Using the combined analyses of 16S rRNA, *recA*, *atpD*, *glnII*, *nifH*, and *nodC* gene sequences, we showed that native rhizobial strains were closely related to *R. tropici*, with one of them representing a potential novel lineage. Native rhizobial strains caused significant increases in root and shoot weight, total C and N contents, and showed greater N_2_-fixation rates and lower emissions of N_2_O both in free-living and symbiotic conditions compared to the non-native rhizobium from a commercial inoculant. The native rhizobial strains isolated in this study could constitute the basis for development of microbial-based inoculants to improve the growth of sunnhemp.

## 5. Materials and Methods

### 5.1. Sample Collection and Rhizobia Trapping Experiment

Bulk soil samples (0–15 cm) were collected from twenty-four random locations within the row middles of two commercial citrus orchards (denoted as A and B) without previous history of CCs in South Florida (USA) (Supplementary Figure S1). Location of the sampling sites and the main physicochemical properties of the soils are presented in Supplementary Table S1. At the time of sampling, row middle soils were naturally covered by diverse weed species such as guinea grass (*Megathyrsus maximus*), goosegrass (*Eleusine indica*), Spanish needles (*Bidens* sp.), globe sedge (*Cyperus globulosus*), *Panicum* sp., and *Lepidium virginicum*. For all citrus orchard soils collected, plant residues were removed, and soil samples were pooled together (about 1 kg of soil was collected in total for each location), kept on ice, and brought to the laboratory. A commercial inoculant (Guard-N, Verdesian, Cary, NC, USA) containing *B. japonicum*, *Bradyrhizobium* sp., *R. leguminorsarum* sv. viceae, and *R. leguminosarum* sv. phaseoli, which is commonly used by growers planting legume CCs in fruit tree orchards in Florida, was used in this study. Treatments were named COA (soil from citrus orchard A), COB (soil from citrus orchard B), and MI (commercial rhizobial inoculant).

Sunnhemp seeds (*C. juncea* L.) were surface sterilized with 99.9% ethanol for 1 min, followed by thorough washing in sterile distilled water. The seeds were then placed in Petri dishes containing 1% water agar and allowed to germinate at 28 °C in the dark for 2 days. Seedlings (3 per pot) were planted in 2 kg pots (12.5 cm diameter × 30 cm height) containing sterile vermiculite and independently inoculated with 1 mL of soil solutions (COA or COB), prepared by homogenization of 1 g of soil in 9 mL of sterile saline solution (0.9% NaCl). The MI was applied following the manufacturer’s instructions. Uninoculated plants were used as a negative control. Six replicate pots per treatment were assayed. The pots were placed in a growth chamber under controlled conditions (28/22 °C day/night; 16/8 h light/dark cycle), and the plants were fertilized every 5 days with a sterile N-free nutrient solution [52]. Plants were harvested at 10% flowering, about 8 weeks after sowing, and nodules were collected for isolation of their interior microbiota and to study their capacity to fix N_2_ and produce N_2_O and N_2_.

### 5.2. Isolation of Bacteria from Nodules and Culture Conditions

Nodules (30 per treatment) were surface sterilized with 99.9% ethanol for 1 min, followed by 3 min in 3% sodium hypochlorite, washed 5 times with sterile deionized water, and then crushed in a drop of sterile water with a sterile glass rod. The resulting suspension was streaked onto Petri dishes containing solid yeast extract mannitol (YEM) medium [53] and incubated at 28 °C for 14 days. To ensure complete external disinfection, some of the disinfected nodules were incubated in the same medium, and no growth was found. After incubation, all colony-forming units (CFUs) were observed on an inverted microscope to select rhizobial strains and representative colonies of the morphologies of potential non-rhizobial strains.

### 5.3. DNA Isolation and Quantification

Bacterial genomic DNA was obtained after growth of the cells in liquid YEM medium as described by Mellal et al. [54]. The supernatant containing the DNA was recovered and kept at −20 °C until use. DNA concentration was measured using the Qubit^®^ dsDNA HS assay kit (Thermo Fisher Scientific, Waltham, MA, USA).

### 5.4. REP-PCR Fingerprinting

Repetitive extragenic palindromic polymerase chain reactions (REP-PCR) were performed as described previously [55]. A database was created with the REP-PCR patterns of all isolates, and a distance matrix was constructed using Jaccard’s similarity coefficient. Then, a dendrogram was built using the unweighted pair group with arithmetic mean (UPGMA) using the Quantity One analysis software (Bio Rad, Hercules, Clearwater, FL, USA).

### 5.5. PCR Amplifications

The fD1 and rD1 universal primers were used to amplify the 16S rRNA gene [56]. The primer pairs glnII 12F and glnII 689R, atpD 255F and atpD 782R, and recA 41F and recA 640R were used for amplification of the *glnII*, *atpD*, and *recA* housekeeping genes, respectively [57]. The combination of housekeeping genes *recA* + *glnII* + *atpD* and *recA* + *glnII* were selected for assessing the evolutionary genetics of the *Rhizobium* and *Bradyhizobium* species because they are known to give the best relative performance [58,59]. Moreover, the housekeeping genes chosen have available sequences in Genebank for the type strains of all the described species of *Rhizobium* and *Bradyrhizobium*. Amplification of the symbiotic *nodC* gene was done using primers nodCF and nodCI [11], whereas the *nifH* gene involved in N_2_-fixation was amplified using the primers nifHF and nifHR [49]. The QIAquick PCR Purification Kit was used to purify the PCR products (Qiagen, Redwood City, CA, USA). The products were analyzed with a 3130 × l automatic sequencer at the sequencing facilities of MCLAB (Molecular Cloning Laboratories, San Francisco, CA, USA). Sequences were compared with those from GenBank using the BLASTN tool and the EzBioCloud database and aligned using the Clustal W program [60]. Phylogenetic trees were built using the maximum likelihood (ML) [61] analysis, and distances were calculated according to Kimura’s two-parameter model [62]. MEGA 7.0 was used for the phylogenetic analyses [63]. Accession numbers of the 16S rRNA, *recA*, *atpD*, *glnII*, *nodC*, and *nifH* sequences of the strains used in this study are shown in the phylogenetic trees.

### 5.6. Plant Nodulation Tests

To confirm the nodulation capacity of the isolates, infectivity tests were conducted. Seeds of *C. juncea* species were surface sterilized as described above. Seedlings (3 per pot) were planted in 2 kg pots (12.5 cm diameter × 30 cm height) containing sterile vermiculite as substrate and independently inoculated at sowing with 1 mL of a bacterial strain (~10^8^ cells mL^−1^). Six replicate pots per strain were assayed. Uninoculated plants (six replicates) were used as a negative control. The plants were irrigated with a sterile N-free nutrient solution [52] every 5 days, grown in a growth chamber under controlled conditions (28/22 °C day/night; 16/8 h light/dark cycle), and harvested at 10% flowering. Nodules were collected and their interior microbiota isolated as described above. REP-PCR was performed to confirm the presence of the inoculated bacteria in the nodules by comparing their REP-PCR pattern with that of the strains used for inoculating the plants using the Quantity One analysis software (Bio Rad, Hercules, Clearwater, FL, USA).

### 5.7. Acetylene Reduction Activity (ARA)

The nitrogenase activity of the isolates and nodules (approximately 20 nodules) was assessed by the ARA assay as previously described [64]. Briefly, cells were inoculated in liquid YEM medium without yeast extract and incubated at 28 °C for 10 days in 100 mL glass jars closed with serum rubber caps to allow injection and withdrawal of gas samples. Then, 10% of the internal atmosphere of each jar was removed and replaced with the same volume of acetylene. N_2_-fixation activity of nodules was assayed as described above by placing approximately 20 nodules in 12 mL glass vials. Gas samples (5 mL) were taken every 12 h for 10 days and analyzed for ethylene production using a gas chromatograph (Model GC 8A, Shimadzu Japan) equipped with a flame ionization detector and HayeSep N column (80–100 mesh). The acetylene reduction rate from free-living cells and nodules was calculated by measuring the increase of ethylene (C_2_H_4_) production inside the vial’s headspace determined in the lineal range [64].

### 5.8. N_2_O and N_2_ Emissions

The ability of the isolates and nodules to produce and reduce N_2_O was assayed as described by Castellano-Hinojosa et al. [65]. Cells were inoculated in liquid YEM medium supplemented with 10 mM NO_3_^−^ and incubated at 28 °C for 3 days in 100 mL glass jars closed with serum rubber caps to allow injection and withdrawal of gas samples and evacuated with pure argon to ensure N_2_-free conditions [14]. Samples were prepared in duplicate. Then, 10% of the internal atmosphere of half of the jars was removed and substituted with acetylene. Gas samples (5 mL) were taken every 12 h and analyzed using a SRI 8610C greenhouse gas chromatograph (SRI Instruments, Las Vegas, NV, USA) equipped with two 1.8 m HayeSep D columns in vent valve configuration and an electron-capture detector to analyze N_2_O concentrations. Calculation of the difference in N_2_O production in the presence and absence of acetylene was used to estimate N_2_ production. N_2_O and N_2_ emissions from nodules were assayed as described above by placing 20 nodules in 12 mL glass vials. After incubation for 2 and 4 h at 28 °C, gas samples (1 mL) were extracted from the tubes for N_2_O analysis as described above.

### 5.9. Plant Inoculation Assays

Soil samples (0–15 cm) were taken from 24 random locations within the row middles of a citrus orchard at the University of Florida Southwest Florida Research and Education Center (Immokalee, FL, USA). The soil had a sandy texture and the following characteristics: pH (in water) 7.1, 93.8% sand, 5.0% silt, 1.2% clay, 1.2% organic matter, 3.4 mg kg^−1^ ammonium (NH_4_^+^), 5.4 mg kg^−1^ NO_3_^−^. Plant residues were removed, and soil samples were pooled together. The soil was used to fill 2 kg pots (12.5 cm diameter × 30 cm height), which were placed in a greenhouse under controlled conditions and watered daily for 5 min using a drip irrigation system (2 L/hour). Seeds of *C. juncea* species (3 per pot) were planted, and the plants were grown for 8 weeks until about 10% flowering. Five bacterial strains were selected for plant inoculation assays based on their ability to nodulate sunnhemp plants. Seedlings were independently inoculated at sowing with 1 mL of the corresponding bacterial strains (~10^8^ cells mL^−1^). Six replicate pots per strain were assayed. Uninoculated plants were used as a control. Shoot and root dry weight were determined on samples that had been dried at 60 °C for 48 h and total carbon (C) and total nitrogen (N) in the shoots were determined using a Leco TruSpec CN Elemental Analyser (Saint Joseph, MI, USA). The nodules on the roots were picked and counted. A subsample of the nodules was used for N_2_-fixation, N_2_O, and N_2_ analyses as described above.

### 5.10. Statistical Analysis

Data analysis was performed using the R software version 4.1.2 (http://www.rproject.org/ (accessed on 10 October 2021)). Normality and homoscedasticity assumptions were tested by using Shapiro–Wilk and Bartlett’s tests, respectively. Significant differences among treatments were assessed with one-way analysis of variance (ANOVA) and Tukey’s HSD post hoc tests.

## Figures and Tables

**Table 1:** Identification of rhizobial strains isolated from sunnhemp (*Crotalaria juncea* L.) based on the amplification of the 16S rRNA gene. Treatments were named: COA (soil from citrus orchard A), COB (soil from citrus orchard B), and MI (commercial rhizobial inoculant). * Strains in bold were chosen for subsequent phylogenetic, in vitro, and in planta analyses.

Strain *	REP-PCR Pattern	Closest Relative Species According to 16S rRNA Gene Sequence	% Similarity (According to EzTaxon-e)
COA1, COA2	1	*Bacillus paranthracis*	99.95
**COA3**	2	*Rhizobium* sp./*R. tropici*	99.87
COA4	3	*Bacillus paramycoides*	100
COA5	4	*Herbaspirillum seropedicae*	99.85
**COA6**	5	*Rhizobium* sp./*R. tropici*	99.78
CO7, COA8	6	*Bacillus cereus*	100
COA9, COA10, COA11	7	*Bacillus nitratoreducens*	99.84
COA12	8	*Bacillus* sp.	99.10
COA13	9	*Paenibacillus polymyxa*	99.58
COB1	10	*Burkholderia cepacia*	99.90
COB2, COB7	11	*Burkholderia territori*	99.85
COB3, COB9	12	*Bacillus megaterium*	100
COB4, COB10	13	*Herbaspirillum robiniae*	99.90
**COB5**	14	*Rhizobium* sp./*R. tropici*	99.85
**COB6**	15	*Rhizobium* sp./*R. tropici*	99.82
COB8, COB11	16	*Paenarthrobacter nicotinovorans*	99.84
COB12, COB13	17	*Bacillus* sp.	100
COB14, COB15, COB17	18	*Bacillus aryabhattai*	99.95
COB16, COB18, COB19	19	*Paenibacillus polymyxa*	99.85
MI1, MI3, MI4	20	*Bacillus* sp.	99.85
MI2, MI5	21	*Paenibacillus* sp.	99.70
MI6, MI10, MI15	22	*Burkholderia cepacia*	99.84
MI7, MI8, MI13	23	*Bacillus* sp.	99.90
MI9, MI12	24	*Bacillus* sp.	100
MI11, MI14	25	*Bacillus aryabhattai*	100
**MI13**	26	*Bradyrhizobium* sp.	99.90

**Table 2:** N_2_-fixation, nitrous oxide (N_2_O), and dinitrogen (N_2_) rates of rhizobial strains in free-living cells (A) and nodules (B) of sunnhemp (*Crotalaria juncea* L.) inoculated with the studied rhizobial strains. Values represent the mean ± standard error. For each analysis, numbers in a column followed by the same letter are not significantly different according to one-way ANOVA test (Tukey’s HSD, *p* < 0.05).

A.			
	Free-Living Cells		
Rhizobacterial Strain	N_2_-Fixation (nmol C_2_H_4_ h^−1^ mL^−1^)	N_2_O Production (nmol N_2_O h^−1^ mL^−1^)	N_2_ Production (nmol N_2_O h^−1^ mL^−1^)
COA3	18.9 ± 2.4 a	23.9 ± 4.2 b	18.9 ± 1.0 a
COA6	16.9 ± 2.2 a	25.9 ± 3.7 b	18.1 ± 1.2 a
COB5	10.9 ± 1.0 b	27.9 ± 3.1 b	17.9 ± 1.1 a
COB6	10.5 ± 1.2 b	24.9 ± 3.3 b	17.7 ± 1.2 a
MI13	11.9 ± 3.8 b	45.9 ± 3.2 a	16.9 ± 1.2 a
**B.**			
	**Nodules**		
**Rhizobacterial Strain/Treatment**	**N_2_-Fixation (nmol l^−1^ C_2_H_4_ h^−1^ g Nodule Dry Weight^−1)^**	**N_2_O Production (nmol l^−1^ N_2_O h^−1^ g Nodule Dry Weight^−1^)**	**N_2_ Production (nmol l^−1^ N_2_O h^−1^ g Nodule Dry Weight^−1^)**
COA3	152.7 ± 11.8 a	12.7 ± 5.6 b	10.9 ± 0.8 a
COA6	162.7 ± 17.6 a	11.8 ± 6.7 b	9.8 ± 0.6 a
COB5	111.7 ± 14.7 b	12.7 ± 7.8 b	10.8 ± 0.9 a
COB6	120.7 ± 12.9 b	15.8 ± 6.9 b	12.9 ± 0.6 a
MI13	85.7 ± 10.8 c	41.7 ± 11.5 a	15.1 ± 0.4 a

**Table 3:** Number of nodules, root dry weight, shoot dry weight, total C, and total N (%) of sunnhemp (*Crotalaria juncea* L.) inoculated with different rhizobial strains. Uninoculated plants were used as a control. Numbers in a column followed by the same letter are not significantly different according to one-way ANOVA test (Tukey’s HSD, *p* < 0.05).

Rhizobacterial Strain	Number of Nodules per Plant	Root Dry Weight (g)	Shoot dry Weight (g)	Total N (mg/g)	Total C (mg/g)
COA3	65 ± 10 a	45.8 ± 5.4 a	19.2 ± 1.4 a	43.6 ± 1.2 a	399.1 ± 3.2 a
COA6	69 ± 12 a	42.8 ± 4.6 a	18.2 ± 1.3 a	45.1 ± 1.9 a	398.1 ± 2.9 a
COB5	42 ± 11 b	28.8 ± 7.1 b	14.8 ± 1.1 b	36.2 ± 0.6 b	390.6 ± 1.6 b
COB6	44 ± 10 b	34.7 ± 4.5 b	15.5 ± 1.3 b	34.6 ± 1.1 b	391.5 ± 1.1 b
MI13	48 ± 12 b	25.3 ± 3.5 c	12.3 ± 1.0 c	29.1 ± 0.6 c	384.6 ± 1.6 c
Negative control	-	20.8 ± 2.1 c	11.6 ± 1.0 c	20.6 ± 0.5 d	360.6 ± 1.5 d

## Data Availability

The data presented in this study are available on request from the corresponding author.

## Supplementary Materials

The following supporting information can be downloaded at: https://www.mdpi.com/article/10.3390/plants11223011/s1, Table S1. Soil physicochemical properties of the studied sites. Table S2. Identification of strains from nodules of sunnhemp (*Crotalaria juncea* L.). Closest relative type strains of the genera *Rhizobium* and *Bradyrhizobium* on the basis of different targeted genes. Identity values are shown in brackets. Table S3. N_2_-fixation, nitrous oxide (N_2_O), and dinitrogen (N_2_) rates of rhizobial strains from nodules of sunnhemp (*Crotalaria juncea* L.) inoculated with soil from commercial orchards A and B (COA and COB, respectively) and the microbial inoculant (MI). Values represent the mean ± standard error. For each gas analysis, numbers in a column followed by the same letter are not significantly different according to one-way ANOVA test (Tukey’s HSD, *p* < 0.05). Figure S1. Map showing the location of the sampling sites within Citrus orchard A and Citrus orchard B. Figure S2. ML phylogenetic tree based on partial 16S rRNA sequences of strains from nodules of sunnhemp (*Crotalaria juncea* L.) and phylogenetically related species within the genus Rhizobium. The analysis was based on 1420 nucleotides. Isolates are denoted in bold. Bootstrap values are indicated as percentages derived from 1000 replications. Values lower than 70 are not shown. Bar, 1 nucleotides substitution per 100 nucleotides. The tree is rooted with *Bradyrhizobium* diazoefficiens USDA10T. Figure S3. ML phylogenetic tree based on partial 16S rRNA sequences of strains from nodules of sunnhemp (*Crotalaria juncea* L.) and phylogenetically related species within the genus *Bradyrhizobium*. The analysis was based on 1415 nucleotides. Isolates are denoted in bold. Bootstrap values are indicated as percentages derived from 1000 replications. Values lower than 70 are not shown. Bar, 1 nucleotides substitution per 100 nucleotides. The tree is rooted with Bosea thiooxidans DSM 9653T. Figure S4. ML phylogenetic tree based on nifH sequences of strains from nodules of strains from nodules of sunnhemp (*Crotalaria juncea* L.) and phylogenetically related species within the genus Rhizobium. The analysis was based on 340 nucleotides. Isolates are denoted in bold. Bootstrap values are indicated as percentages derived from 1000 replications. Values lower than 70 are not shown. Bar, 2 nucleotide substitution per 100 nucleotides. Figure S5. ML phylogenetic tree based on nifH sequences of strains from nodules of strains from nodules of sunnhemp (*Crotalaria juncea* L.) and phylogenetically related species within the genus Bradyhizobium. The analysis was based on 420 nucleotides. Isolates are denoted in bold. Bootstrap values are indicated as percentages derived from 1000 replications. Values lower than 70 are not shown. Bar, 2 nucleotide substitution per 100 nucleotides.
