# Supplementary material for: Native Rhizobia Improve Plant Growth, Fix N2, and Reduce Greenhouse Emissions of Sunnhemp More than Commercial Rhizobia Inoculants in Florida Citrus Orchards
Source: Plants (Basel). 2022 Nov 8;11(22):3011. doi: 10.3390/plants11223011 (PMC9695096; doi:10.3390/plants11223011)
Supplement: Supplementary file 1 [file plants-11-03011-s001.zip › plants-1993753-supplementary.pdf]

Supplementary Material

Native Rhizobia Improve Plant Growth, Fix N2, and Reduce Greenhouse Emissions of Sunnhemp More than Commercial Rhizobia Inoculants in Florida Citrus Orchards

Authors: Antonio Castellano-Hinojosa*, Christoph Mora, and Sarah L. Strauss

Affiliations: Southwest Florida Research and Education Center, Department of Soil and Water Sciences, Institute of Food and Agricultural Sciences, University of Florida, 2685 State Rd 29N, Immokalee, FL, 34142, USA

*Corresponding author:

Antonio Castellano-Hinojosa

Southwest Florida Research and Education Center, Department of Soil and Water Sciences, Institute of Food and Agricultural Sciences, University of Florida, 2685 State Rd 29N, Immokalee, FL, 34142, USA

antonio.castella@ufl.edu

Table S1. Soil physicochemical properties of the studied sites.

	Citrus orchard A	Citrus orchard B
Soil texture	Sandy	Sandy
Sand (%)	93.5	95.2
Silt (%)	5.5	4.0
Clay (%)	1	0.8
Soil organic matter (%)	2.2	1.1
Ammonium (mg/kg)	6.7	2.4
Nitrate (mg/kg)	8.4	2.6
Soil pH	7.4	7.1

Table S2. Identification of strains from nodules of sunnhemp (*Crotalaria juncea* L.). Closest relative type strains of the genera *Rhizobium* and *Bradyrhizobium* based on different targeted genes. Identity values are shown in brackets.

	Closest relative type strains (Identity values are shown in brackets)					
	Targeted gene					
Rhizobacterial strain	16S rRNA	*recA*	*glnII*	*atpD*	*nifH*	*nodC*
COA3	*R. tropici* CIAT 899^T (99.06%), *R. freirei* PRF 81^T (99.25%), and *R. hainanense* CCBAU 57015^T (99.7%)	*R. tropici* CIAT 899^T (95.7%)	*R. tropici* CIAT 899^T (96.7%)	*R. tropici* CIAT 899^T (95.9%)	*R. tropici* CIAT 899^T (99.2%), *R. multihospitium* CCBAU 83401^T (99.2%), and *R. lusitanum* P1-7^T (99.2%)	*R. tropici* CIAT 899^T (99.1%)
COA6	*R. tropici* CIAT 899^T (99.43%), *R. freirei* PRF 81^T (99.33%), and *R. hainanense* CCBAU 57015^T (99.43%)	*R. tropici* CIAT 899^T (99.6%)	*R. tropici* CIAT 899^T (99.7%)	*R. tropici* CIAT 899^T (99.7%)	*R. tropici* CIAT 899^T (99.2%), *R. multihospitium* CCBAU 83401^T (99.2%), and *R. lusitanum* P1-7^T (99.2%)	*R. tropici* CIAT 899^T (99.2%)
COB5	*R. tropici* CIAT 899^T (99.42%), *R. freirei* PRF 81^T (99.32%), and *R. hainanense* CCBAU 57015^T (99.41%)	*R. tropici* CIAT 899^T (99.6%)	*R. tropici* CIAT 899^T (99.8%)	*R. tropici* CIAT 899^T (99.6%)	*R. tropici* CIAT 899^T (99.2%), *R. multihospitium* CCBAU 83401^T (99.2%), and *R. lusitanum* P1-7^T (99.2%)	*R. tropici* CIAT 899^T (99.2%)
COB6	*R. tropici* CIAT 899^T (99.52%), *R. freirei* PRF 81^T (99.38%), and *R. hainanense* CCBAU 57015^T (99.38%)	*R. tropici* CIAT 899^T (99.6%)	*R. tropici* CIAT 899^T (99.7%)	*R. tropici* CIAT 899^T (99.6%)	*R. tropici* CIAT 899^T (99.2%), *R. multihospitium* CCBAU 83401^T (99.2%), and *R. lusitanum* P1-7^T (99.2%)	*R. tropici* CIAT 899^T (99.2%)
MI13	*B. japonicum* USDA6^T (99.85%), and *B. liaoningense* LMG18230^T (99.93%)	*B. japonicum* USDA6^T (99.5%)	*B. japonicum* USDA6^T (99.6%)	-	*B. daqingense* CCBAU 15774^T (99.9%), *B. huanghuaihainense* CCABU 23303^T (99.9%), *B. ottawaense* OO99^T, *B. liaoningense* LMG 18230^T (99.9%), and *B. japonicum* USDA6^T (99.9%)	*B. yuanmingense* CCBAU10071^T (99.9%)

Table S3. N_2-fixation, nitrous oxide (N_2O), and dinitrogen (N_2) rates of rhizobial strains from nodules of sunnhemp (*Crotalaria juncea* L.) inoculated with soil from commercial orchards A and B (COA and COB, respectively) and the microbial inoculant (MI). Values represent the mean ± standard error. For each gas analysis, numbers in a column followed by the same letter are not significantly different according to one-way ANOVA test (Tukey's HSD, $p < 0.05$).

	Nodules		
Treatment	N_2-fixation (nmol C_2H_4 h^{-1} mL^{-1})	N_2O production (nmol N_2O h^{-1} mL^{-1})	N_2 production (nmol N_2O h^{-1} mL^{-1})
COA	102.7 ± 15.8a	18.7 ± 5.6b	1.5 ± 0.8a
COB	140.4 ± 28.1a	22.8 ± 6.2b	1.0 ± 0.6a
MI	48.2 ± 16.6b	39.7 ± 8.5a	1.1 ± 0.9a

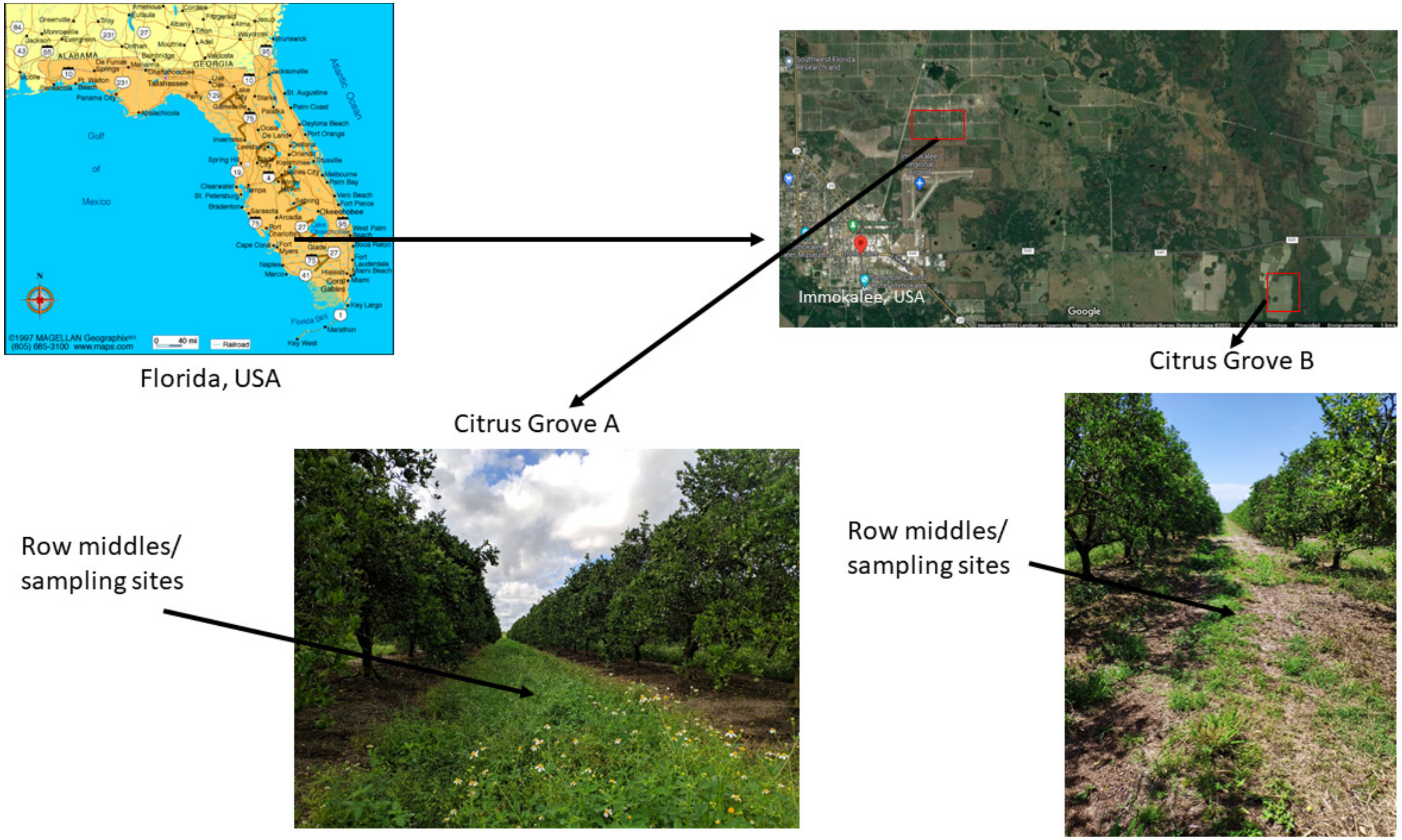

Figure S1. Map showing the location of the sampling sites within Citrus orchard A and Citrus orchard B.

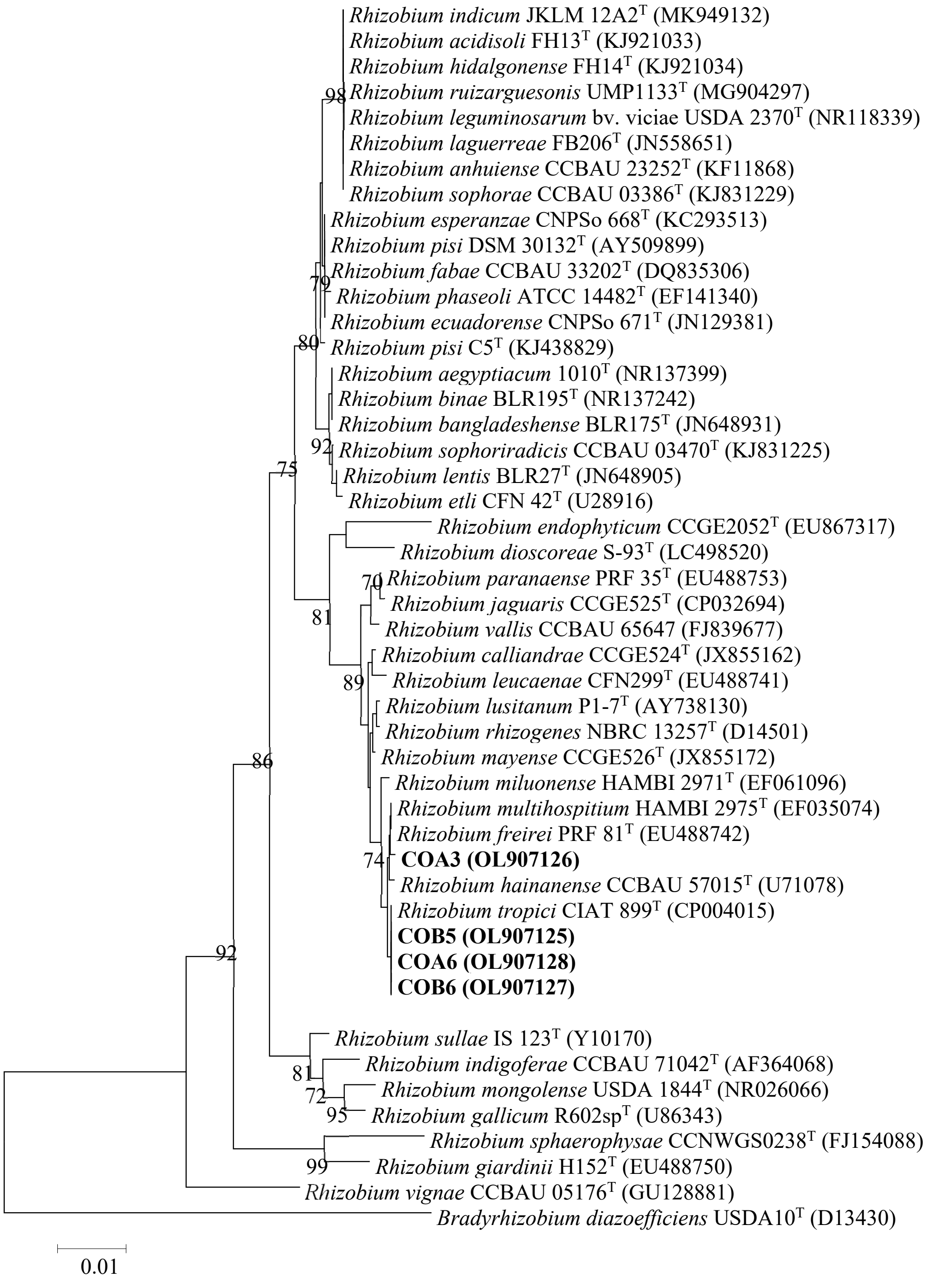

Figure S2. ML phylogenetic tree based on partial 16S rRNA sequences of strains from nodules of sunnhemp (*Crotalaria juncea* L.) and phylogenetically related species within the genus *Rhizobium*. The analysis was based on 1420 nucleotides. Isolates are denoted in bold. Bootstrap values are indicated as percentages derived from 1000 replications. Values lower than 70 are not shown. Bar, 1 nucleotides substitution per 100 nucleotides. The tree is rooted with *Bradyrhizobium* diazoefficiens USDA10^{T}.

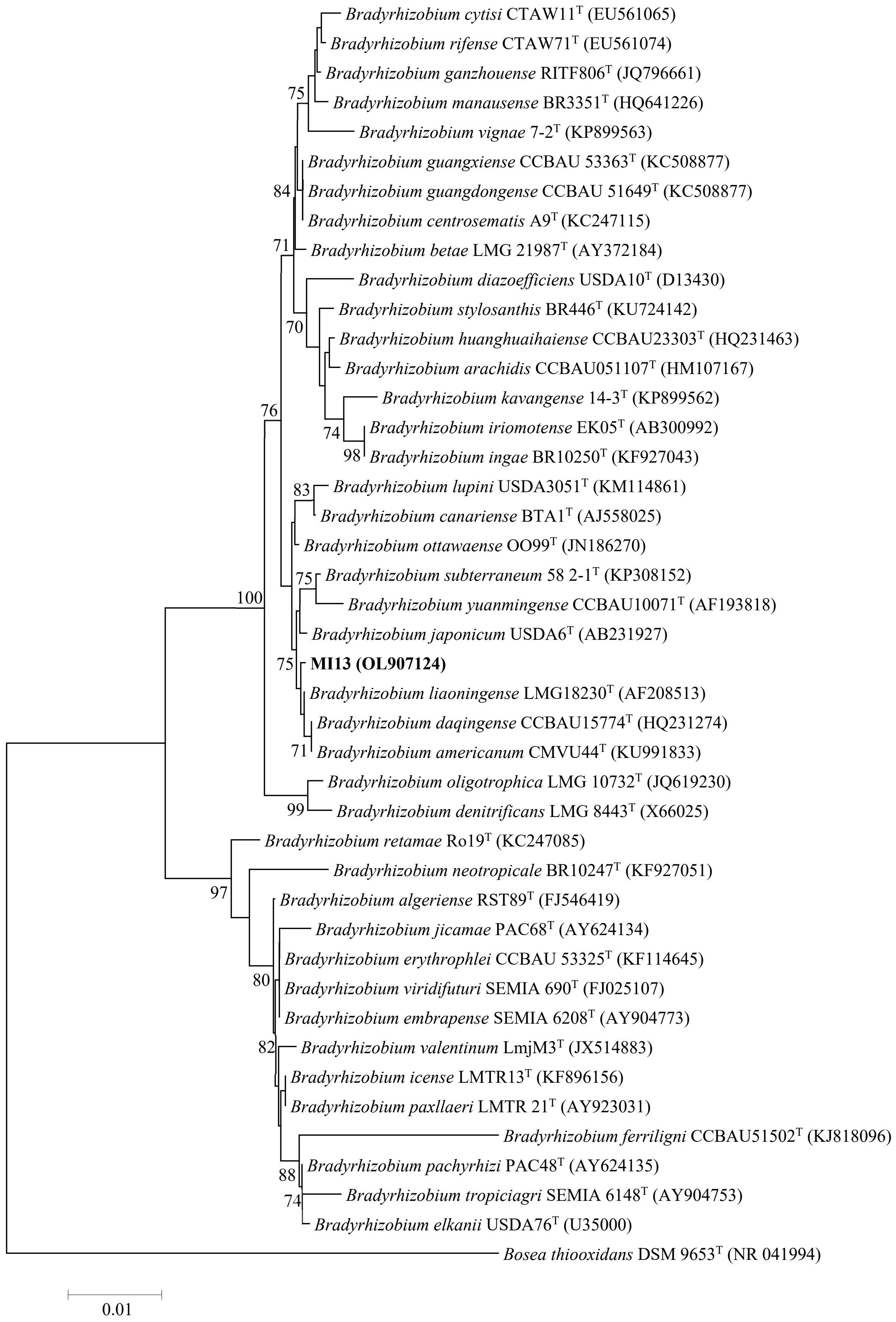

Bradyrhizobium cytisi CTAW11T (EU561065)
Bradyrhizobium rifense CTAW71T (EU561074)
Bradyrhizobium ganzhouense RITF806T (JQ796661)
Bradyrhizobium manausense BR3351T (HQ641226)
Bradyrhizobium vignae 7-2T (KP899563)
Bradyrhizobium guangxiense CCBAU 53363T (KC508877)
Bradyrhizobium guangdongense CCBAU 51649T (KC508877)
Bradyrhizobium centrosematis A9T (KC247115)
Bradyrhizobium betae LMG 21987T (AY372184)
Bradyrhizobium diazoefficiens USDA10T (D13430)
Bradyrhizobium stylosanthis BR446T (KU724142)
Bradyrhizobium huanghuaihaiense CCBAU23303T (HQ231463)
Bradyrhizobium arachidis CCBAU051107T (HM107167)
Bradyrhizobium kavangense 14-3T (KP899562)
Bradyrhizobium iriomotense EK05T (AB300992)
Bradyrhizobium ingae BR10250T (KF927043)
Bradyrhizobium lupini USDA3051T (KM114861)
Bradyrhizobium canariense BTA1T (AJ558025)
Bradyrhizobium ottawaense OO99T (JN186270)
Bradyrhizobium subterraneum 58 2-1T (KP308152)
Bradyrhizobium yuanmingense CCBAU10071T (AF193818)
Bradyrhizobium japonicum USDA6T (AB231927)
MI13 (OL907124)
Bradyrhizobium liaoningense LMG18230T (AF208513)
Bradyrhizobium daqingense CCBAU15774T (HQ231274)
Bradyrhizobium americanum CMVU44T (KU991833)
Bradyrhizobium oligotrophica LMG 10732T (JQ619230)
Bradyrhizobium denitrificans LMG 8443T (X66025)
Bradyrhizobium retamae Ro19T (KC247085)
Bradyrhizobium neotropicale BR10247T (KF927051)
Bradyrhizobium algeriense RST89T (FJ546419)
Bradyrhizobium jicamae PAC68T (AY624134)
Bradyrhizobium erythrophlei CCBAU 53325T (KF114645)
Bradyrhizobium viridifuturi SEMIA 690T (FJ025107)
Bradyrhizobium embrapense SEMIA 6208T (AY904773)
Bradyrhizobium valentinum LmjM3T (JX514883)
Bradyrhizobium icense LMTR13T (KF896156)
Bradyrhizobium paxllaeri LMTR 21T (AY923031)
Bradyrhizobium ferriligni CCBAU51502T (KJ818096)
Bradyrhizobium pachyrhizi PAC48T (AY624135)
Bradyrhizobium tropiciagri SEMIA 6148T (AY904753)
Bradyrhizobium elkanii USDA76T (U35000)
Bosea thiooxidans DSM 9653T (NR 041994)
75
84
71
70
76
74
98
83
75
100
75
71
99
97
80
82
88
74
0.01

Figure S3. ML phylogenetic tree based on partial 16S rRNA sequences of strains from nodules of sunnhemp (*Crotalaria juncea* L.) and phylogenetically related species within the genus *Bradyrhizobium*. The analysis was based on 1415 nucleotides. Isolates are denoted in bold. Bootstrap values are indicated as percentages derived from 1000 replications. Values lower than 70 are not shown. Bar, 1 nucleotides substitution per 100 nucleotides. The tree is rooted with *Bosea thiooxidans* DSM 9653^{T}.

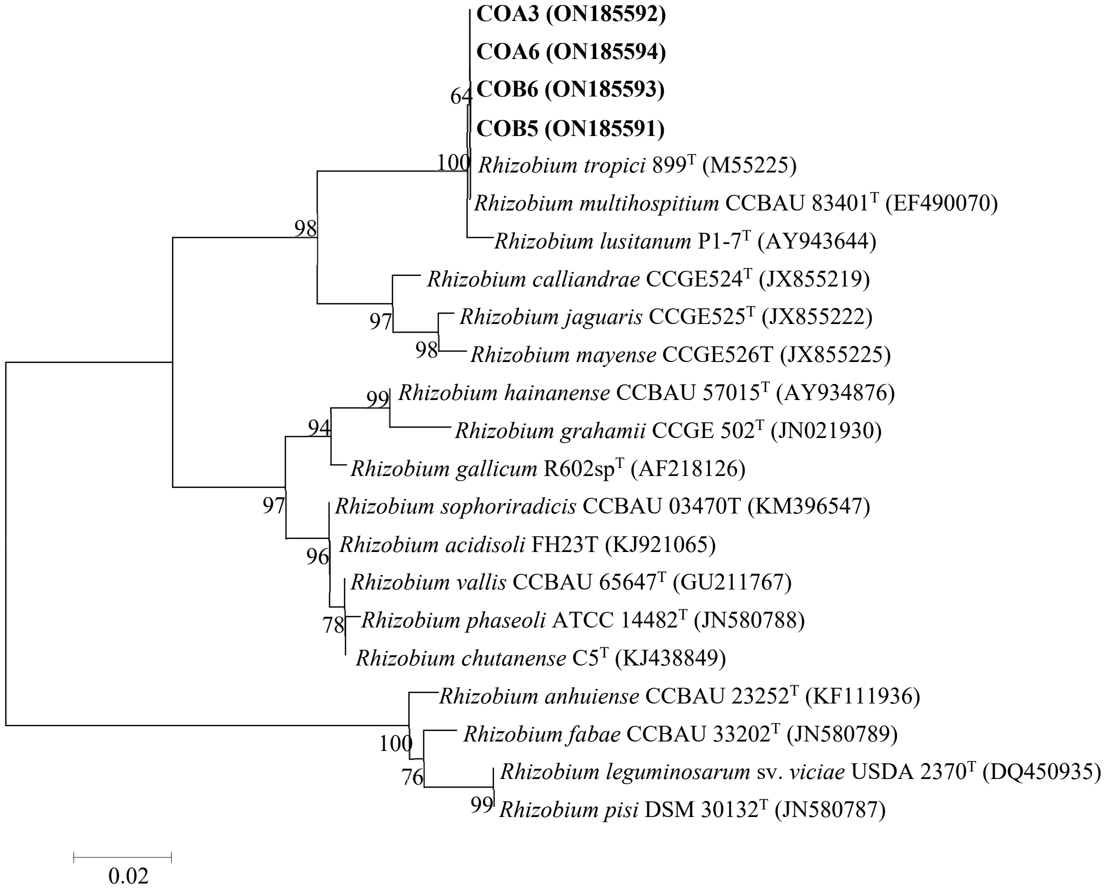

Figure S4. ML phylogenetic tree based on *nifH* sequences of strains from nodules of strains from nodules of sunnhemp (*Crotalaria juncea* L.) and phylogenetically related species within the genus *Rhizobium*. The analysis was based on 340 nucleotides. Isolates are denoted in bold. Bootstrap values are indicated as percentages derived from 1000 replications. Values lower than 70 are not shown. Bar, 2 nucleotide substitution per 100 nucleotides.

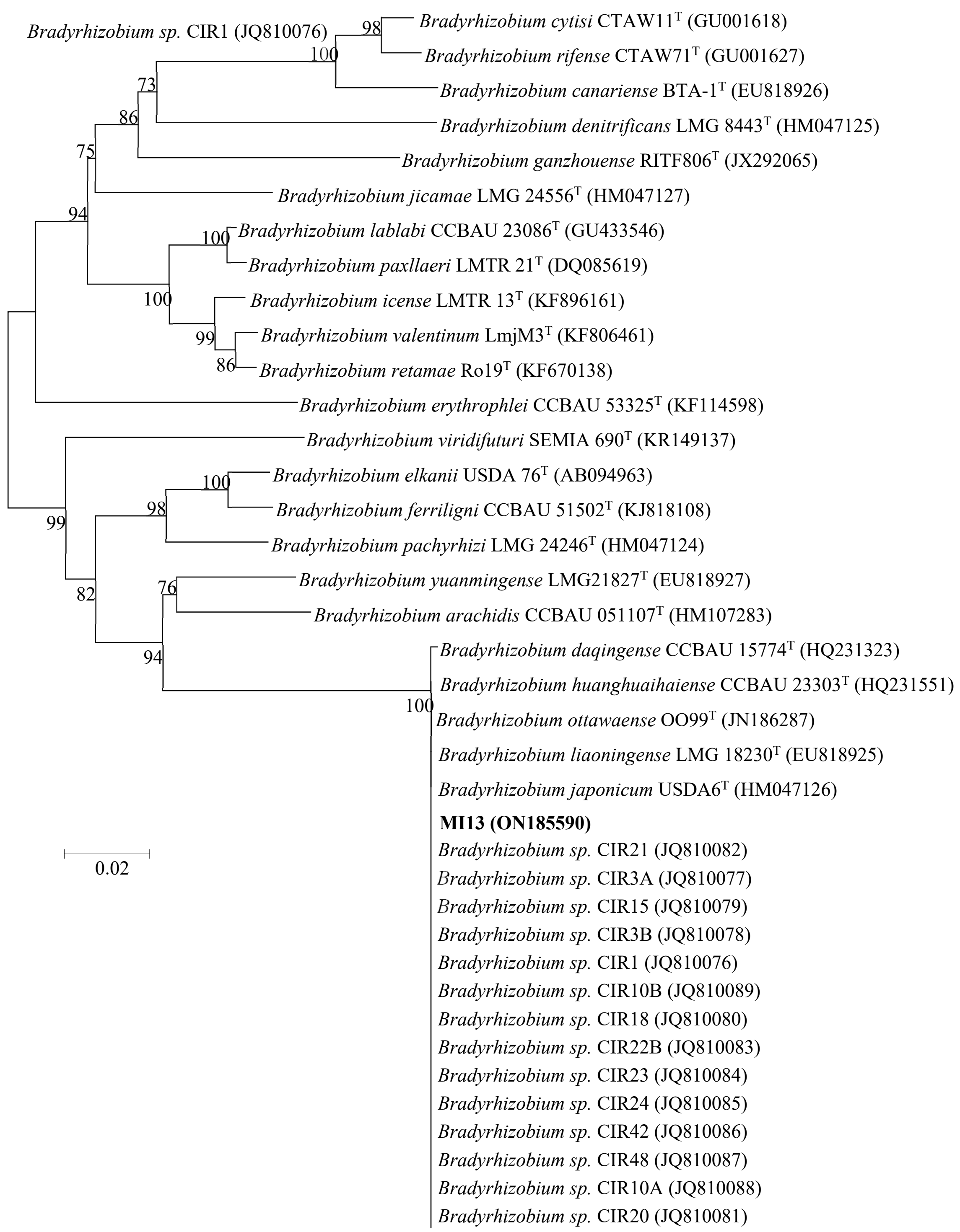

Figure S5. ML phylogenetic tree based on *nifH* sequences of strains from nodules of strains from nodules of sunnhemp (*Crotalaria juncea* L.) and phylogenetically related species within the genus *Bradyhizobium*. The analysis was based on 420 nucleotides. Isolates are denoted in bold. Bootstrap values are indicated as percentages derived from 1000 replications. Values lower than 70 are not shown. Bar, 2 nucleotide substitution per 100 nucleotides.
